# Integrated transcriptomic and proteomic analysis of exogenous abscisic acid regulation on tuberous root development in *Pseudostellaria heterophylla*

**DOI:** 10.3389/fnut.2024.1417526

**Published:** 2024-07-05

**Authors:** Chongmin Wang, Jiaotong Yang, Qi Pan, Panpan Zhu, Jun Li

**Affiliations:** Guizhou University of Traditional Chinese Medicine, Guiyang, China

**Keywords:** abscisic acid, multi-omics analysis, root morphogenesis, gene regulatory networks, biochemical pathways

## Abstract

Abscisic acid (ABA) significantly regulates plant growth and development, promoting tuberous root formation in various plants. However, the molecular mechanisms of ABA in the tuberous root development of *Pseudostellaria heterophylla* are not yet fully understood. This study utilized Illumina sequencing and *de novo* assembly strategies to obtain a reference transcriptome associated with ABA treatment. Subsequently, integrated transcriptomic and proteomic analyses were used to determine gene expression profiles in *P. heterophylla* tuberous roots. ABA treatment significantly increases the diameter and shortens the length of tuberous roots. Clustering analysis identified 2,256 differentially expressed genes and 679 differentially abundant proteins regulated by ABA. Gene co-expression and protein interaction networks revealed ABA positively induced 30 vital regulators. Furthermore, we identified and assigned putative functions to transcription factors (PhMYB10, PhbZIP2, PhbZIP, PhSBP) that mediate ABA signaling involved in the regulation of tuberous root development, including those related to cell wall metabolism, cell division, starch synthesis, hormone metabolism. Our findings provide valuable insights into the complex signaling networks of tuberous root development modulated by ABA. It provided potential targets for genetic manipulation to improve the yield and quality of *P. heterophylla*, which could significantly impact its cultivation and medicinal value.

## 1 Introduction

*Pseudostellaria heterophylla* (Miq.) Pax ex Pax et Hoffm is a plant of the *Caryophyllaceae* family, and its dried tuberous root is an important medicinal material in traditional Chinese medicine, known as tai-zi-shen or hai-er-shen in Chinese. Studies have found that the tuberous root of *P. heterophylla* contains rich medicinal active ingredients such as polysaccharides (1), cyclic peptides (2), and saponins (3). In clinical practice, *P. heterophylla* is a commonly used traditional Chinese medicinal herb known for its efficacy in invigorating the spleen, replenishing qi, moistening the lung and benefiting blood (4). Research has shown that the contents of secondary metabolites in the roots are significantly associated with the species (5). Different types of cortex and xylem cells lead to variations in the distribution of secondary metabolites (6). Therefore, enhancing its medicinal properties by investigating the regulatory mechanisms of *P. heterophylla* tuberous root development is crucial.

Abscisic acid (ABA) is a 15-carbon sesquiterpene molecule identified in the 1960s and an essential regulatory factor in developing tuberous roots (7). In the process of plant tuberous root development, ABA is involved in regulating various physiological processes, including cell division and elongation (8), cell proliferation (9), tissue differentiation (10), root elongation (11), and nutrient accumulation (12–14). The growth, development and accumulation of secondary metabolites in numerous medicinal plants have now been linked to the regulation of ABA, such as perilla (15), ginseng (16), and rehmannia (17). The development of the tuberous root was affected by cell proliferation in both horizontal and vertical directions, which is directly related to the synthesis and transportation of hormones in the tuberous root. Therefore, understanding the regulation of ABA is critical to analyzing the formation of the tuberous root morphology of *P. heterophylla*. However, the role of ABA in *P. heterophylla* tuberous root regulation is still unknown.

The root developmental processes and architecture are accurately controlled by several genetic regulatory factors, including plant hormones, transcription factors (TFs), and peptides (18–20). TFs, a vital category of regulators for root characters, have gained prominent attention and have been extensively identified in various plant species (21, 22). In addition to participating in stress response, ABA is intricately linked to numerous metabolic and cellular biological processes (23). ABA mediates its signal pathway through a dual negative regulatory system. PP2C inhibits the activity of the positive ABA regulator SnRK2, and ABA recognizes PP2C through the receptor, releasing SnRK2 from the inhibited state of PP2C (24). The active SnRK2 kinase phosphorylates and regulates the TF targets involved in the ABA response, thereby initiating the transcription of ABA-responsive genes (25). ABI5 and ABFs are members of the basic leucine zipper (bZIP) family of TFs and play significant roles in the ABA signaling pathway (26–28). With the deepening of research, more and more TFs are involved in response to ABA signals (29), such as MYB (30, 31), bZIP (32, 33), and SBP (34). However, the research on the transcriptional regulatory mechanisms involved in developing *P. heterophylla* tuberous root is poorly understood. Investigating and clarifying the ABA-mediated tuberous root development would be highly beneficial for the future genetic improvement of *P. heterophylla*.

Integrating multi-omics technologies has become a powerful tool for revealing the regulatory network of plant development (35). Previous studies have utilized multi-omics technologies, including transcriptomics, proteomics, and metabolomics, to uncover the dynamic changes and stage-specific variability in gene and protein expression during the development of cassava tubers (36). An integrative multi-omics analysis was used in sweet potatoes to screen the related regulatory pathways of the genes/proteins essential in developing the tuberous roots (37). High-throughput sequencing can reveal dynamic gene expression changes, while proteomics explores protein interaction networks in-depth. Our study integrates transcriptome and proteome analysis to elucidate how ABA influences *P. heterophylla* tuberous root development. By analyzing the critical TFs of differently expressed genes (DEGs), characterization of downstream targets, and exploration of synergistic regulatory mechanisms, aiming to improve agricultural productivity and medicinal applications of *P. heterophylla*. This research deepens our understanding of the molecular mechanisms involved and offers new insights for enhancing the medicinal qualities of *P. heterophylla*, significantly impacting its cultivation and medicinal value enhancement.

## 2 Materials and methods

### 2.1 Plant cultivation and treatment

*P. heterophylla* seeds were sown in soil under low temperatures (10°C) for 45 days. Then, uniformly growing plants were transplanted into artificially prepared nutrient soil (humus: vermiculite: perlite = 3:1:1) for pot cultivation. Then, the plants were treated with water, no hormones added (CK, control group), 15 mg/L ABA, and 2 g/L sodium tungstate (NaW) (38). Each group was replicated thrice, and weekly irrigation was done with 200 mL solutions with or without reagent. Samples were collected 60 days after treatment, and physiological indicators such as the diameter and length of the tuberous roots were measured and analyzed.

### 2.2 Sample preparation and microscopy

The tuberous roots of *P. heterophylla* were divided into head, middle, and tail sections and fixed in FAA solution (70% ethanol, formaldehyde, acetic acid = 16,1,1) at 4°C for 24 h. After fixation, samples were dehydrated through an ethanol series, cleared with xylene, and infiltrated with paraffin. Paraffin-embedded samples were sectioned longitudinally at 5–10 μm using a LEICA RM2016 microtome, fixed on slides, and dried overnight at 37°C. Sections were deparaffinized, stained with safranin and fast green. Then, it is dehydrated, cleared, and mounted with neutral gum. Microscopic examination was performed using an optical microscope (Nikon ECLIPSE), with lignified cell walls and nuclei appearing red and other structures appearing green.

The morphological structure was observed using CaseViewer software. Five regions, including periderm (P), secondary phloem (SP), vascular cambium (VC), secondary xylem (SX), and primary xylem (PX), were photographed. The width of each region was recorded, and the number of cell layers, the longitudinal and transverse width of the cells on the line, and the number and diameter of starch granules within the cells on the line were counted.

### 2.3 RNA-seq analysis

Total RNA was extracted from tuberous root tissue using TRIzol^®^ Reagent (Plant RNA Purification Reagent for plant tissue) according to the manufacturer’s instructions (Invitrogen, Carlsbad, CA, United States), and genomic DNA was removed using DNase I (Takara Bio, Shiga, Japan). Then, the integrity and purity of the total RNA were assessed using a 2,100 Bioanalyser (Agilent Technologies, Inc., SantaClara, CA, United States) and quantified with the ND-2000 (NanoDrop Thermo Scientific, Wilmington, DE, United States). Only high-quality RNA samples (OD260/280 = 1.8–2.2, OD260/230 ≥ 2.0, RIN ≥8.0, 28S:18S ≥1.0, >1 μg) were used for library construction.

The construction of the cDNA library and RNAseq was performed by Shanghai Majorbio Bio-Isarm Technology Co., Ltd. (Shanghai, China). First, mRNA was purified from 12 μg of total RNA from three groups (CK, ABA, NaW) using Oligo(dT) magnetic beads, respectively. The first-strand cDNA was formed via reverse transcription using reverse transcriptase and random hexamer primer using mRNA as a template. These cDNA fragments were ligated with the Illumina paired-end sequencing adaptors. Finally, these libraries were sequenced on a paired-end flow cell using the Illumina Novaseq 6,000 platform.

For *de novo* assembly and annotation, raw paired-end reads were trimmed and quality controlled using SeqPrep^1^ and Sickle.^2^ Clean data were assembled with Trinity, and the assembled transcripts were searched against the National Center for Biotechnology Information (NCBI) protein nonredundant (NR), COG, and KEGG databases using BLASTX to retrieve functional annotations, with a cut-off E-value of less than 1e-5. GO annotations were obtained using BLAST2GO, and metabolic pathway analysis was performed with KEGG. Differential expression analysis was done using DESeq2, with a Q value ≤0.01. Genes with |log_2_FC| > 2 and a Q value ≤0.01 (DESeq2) were considered significantly (DEGs). Functional enrichment analysis for GO and KEGG pathways was performed using Goatools and KOBAS, with a Bonferroni-corrected *p*-value ≤0.05, to identify significantly enriched DEGs compared to the whole transcriptome background.

### 2.4 Proteome analysis

Total proteins were extracted from the tuberous roots of *P. heterophylla* using urea lysis buffer and protease inhibitors. Then, the samples were oscillated three times in a high-throughput tissue grinder for 40 s, lysed on ice for 30 min, and vortexed for 5–10 s every 5 min. The supernatant was collected after centrifugation at 12000 g for 30 min at 4°C. Protein concentration was determined using a bicinchoninic acid (BCA) protein assay kit (Thermo, United States). The absorbance was read at 562 nm.

The samples were fractionated using high pH reverse phase separation to increase protein depth. The peptide samples were re-dissolved with UPLC loading buffer [2% acetonitrile (ammonia to pH 10)] and high pH liquid phase separation was performed using a reverse phase C18 column ACQUITY UPLC BEH C18 Column 1.7 μm, 2.1 mm × 150 mm (Waters, United States). The peptides were separated by an elution gradient (phase A: 2% acetonitrile, pH 10; phase B: 80% acetonitrile, pH 10) at a 200 μL/min flow rate over 48 min. Then, two-dimensional analysis was performed by liquid chromatography–tandem mass spectrometry (Evosep One with Orbitrap Exploris 480 mass spectrometer) according to the standard protocol of Majorbio Bio-Pharm Technology Co. Ltd. (Shanghai, China). Finally, the RAW data files were analyzed using Proteome Discoverer (Thermo Scientific, version 2.4) and compared with the NCBI and UniProt databases. The precursor mass tolerance was set to 20 ppm, and the fragment mass tolerance was set to 0.02 Da. The false discovery rate (FDR) for peptide identification was ≤0.01. Protein identification was supported by at least one unique peptide identification.

The data were analyzed through the free online majorbio cloud platform (cloud.majorbio.com). The thresholds of fold change (up-regulation >1.2 and down-regulation <0.83) and *p*-value <0.05 were used to identify differentially abundant proteins (DAPs). Annotation of all identified proteins was performed using GO^3^ and KEGG pathway.^4^ DAPs were further used for GO and KEGG enrichment analysis.

### 2.5 Clustering analysis of the DEGs and DAPs

The Mfuzz method was applied for clustering analysis on the expression of DEGs, DAPs, and all TFs across various continuous samples. To delve deeper into the biological processes associated with the proteins in each cluster, KEGG function and GO pathway enrichment analyses are conducted for the DEGs and DAPs within each cluster, respectively.

### 2.6 Gene co-expression and protein interaction network analysis

The Pearson Correlation Coefficient (PCC) was used to assess DEGs’ linear correlation. A threshold of 0.95 is selected to identify gene pairs with statistically significant co-expression relationships to construct the gene co-expression network. The STRING database (version 10.5) is employed to construct protein–protein interaction (PPI) networks for various DAPs. The networks are visualized with Cytoscape (v 3.8.2).

### 2.7 Statistical analysis

Statistical analysis and graphing were performed using SPSS v.26.0 and GraphPad Prism v.9.0. A significant difference was declared at the *p* ≤ 0.05 probability level. Three biological replications were maintained throughout the experiment.

## 3 Results

### 3.1 Changes in tuberous root structure

**Figure 1:**
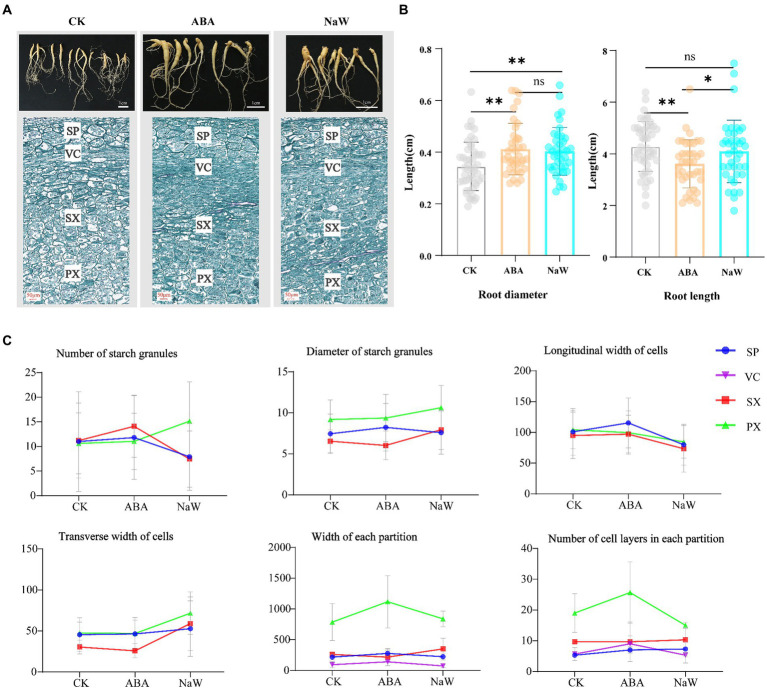
Changes in appearance and morphology of *Pseudostellaria heterophylla* tuberous roots after exogenous ABA treatment. **(A)** Morphology and longitudinal section microstructure of *P. heterophylla* tuberous roots. **(B)** Statistical analysis of *P. heterophylla* tuberous root length and diameter [in comparison to the CK group, *indicates significant differences (*p* < 0.05), **indicates significant differences (*p* < 0.01)]. **(C)** Changes in the microstructure of expansion zone in *P. heterophylla* tuberous roots.

To investigate the effects of exogenous ABA and its biosynthesis inhibitor NaW on tuberous root development in *P. heterophylla*, we characterized the morphology and longitudinal section microstructure of tuberous roots (Figure 1A). The results showed that ABA treatment significantly increases the diameter and shortens the length of tuberous roots (Figure 1B). Subsequently, we examined the changes in the microstructure of *P. heterophylla* tuberous roots in the expansion zone. After the exogenous application of ABA and NaW, the number and size of starch granules, the transverse width of cells, and the longitudinal width of cells changed significantly (Figure 1C; Supplementary Table S1). Particularly, the number of starch granules in the PX and SX regions significantly increased under ABA treatment but significantly decreased under NaW treatment, consistent with changes in longitudinal cell expansion in the same areas, suggesting that starch accumulation may be associated with longitudinal cell expansion and is significantly regulated by ABA. Additionally, exogenous ABA increased the width and cell layer number in the PX region while considerably reducing the transverse width of cells, implying that ABA may regulate the radial development of *P. heterophylla* tuberous roots by enhancing the transverse division ability of cells, thereby promoting tuber swelling.

### 3.2 Transcriptomic analysis overview

Nine cDNA libraries were constructed, and 55.16, 52.73, and 50.59 million raw sequence reads were generated from the CK, ABA, and NaW libraries. After removing low-quality reads and adaptor sequences, 54.51, 52.14, and 50.08 million clean reads with 91.23–91.66% Q30 bases and 44.10–43.60% GC content were obtained, and the clean data of each sample is above 7.31 G (Supplementary Table S2). The resulting *P. heterophylla* transcriptome contained 159,771 transcripts (ranging from 500 to 4,500 bp) and 78,142 unigenes (Supplementary Table S3; Supplementary Figure S1). All unigenes were annotated using Basic Local Alignment Search Tool (BLAST) searches against the following five databases: NCBI NR database (33,904; 43.39% of all identified unigenes), SwissProt (26,120; 33.43%), Protein Families database (Pfam; 24,314; 31.12%), Gene Ontology database (GO; 19,182; 24.55%), and Kyoto Encyclopedia of Genes and Genomes pathway database (KEGG; 15,242; 19.51%), and COG (7,125; 9.12%; Supplementary Figure S2). The NR database provided the most significant number of annotations, and 100,329 unigenes corresponded with sequences from at least one of the public databases, and 3,147 unigenes were annotated to all databases. Among these unigenes, 7,711 were identified as DEGs, including 3,440 (2,274 up- and 1,166 downregulated), 5,729 (3,822 up- and 1907 downregulated), 3,400 (1,237 up- and 2,163 downregulated) and 641 co-expressed in CK_ABA, CK_ NaW and NaW_ABA groups, respectively (Table 1; Supplementary Table S4), which are presented in a volcano plot (Supplementary Figure S3A). These results indicate that a high-quality *P. heterophylla* transcriptome dataset has been obtained through *de novo* assembly.

**Table 1:** Summary of transcripts and proteins detected from RNA and TMT sequence data.

	Transcriptome			Proteome		
	CK_ABA	CK_ NaW	NaW_ABA	CK_ABA	CK_ NaW	NaW_ABA
Unique proteins/genes detected	78,142	78,142	78,142	8,523	8,523	8,523
Significantly DEGs/DAPs	3,440	5,729	3,400	486	801	917
Up-regulated	2,274	3,822	1,237	240	429	455
Down-regulated	1,166	1907	2,163	246	372	462
Shared DEGs/DAPs	641	641	641	84	84	84
Shared DEGs/DAPs (up-regulated)	309	424	232	38	29	50
Shared DEGs/DAPs (downregulated)	332	217	409	46	55	34
Co-regulated DEGs-DAPs	74	115	126	74	115	126
Co-regulated DEGs-DAPs with the same trends	32	82	78	32	82	78
Co-regulated DEGs-DAPs with the opposite trends	42	33	48	42	33	48

### 3.3 Quantitative proteome analysis

In total, 1,075,816 spectra, 249,880 identified spectra, 54,101 peptides, and 8,523 proteins were determined via proteomic analysis (Supplementary Table S3). Regarding protein mass distribution, proteins with molecular weights greater than 1 kDa had a wide range and good coverage, with a maximum distribution area of 1–61 kDa. Peptide quantitative analysis of the proteins showed that protein quantity decreased with increased matching peptides (Supplementary Figure S4). Among these 8,523 proteins, 1,384 were identified as DAPs, including 486 (240 up- and 246 downregulated), 801 (429 up- and 372 down-regulated), 917 (455 up- and 462 down-regulated), and 84 co-expressed proteins in the CK_ABA, CK_NaW, and NaW_ABA groups, respectively (Table 1; Supplementary Table S5), which are presented in a volcano plot (Supplementary Figure S3B). This comprehensive proteomic data enhances our understanding of gene expression outcomes and biological function execution, indicating high-quality data acquisition.

### 3.4 Association analysis of DEGs and DAPs

**Figure 2:**
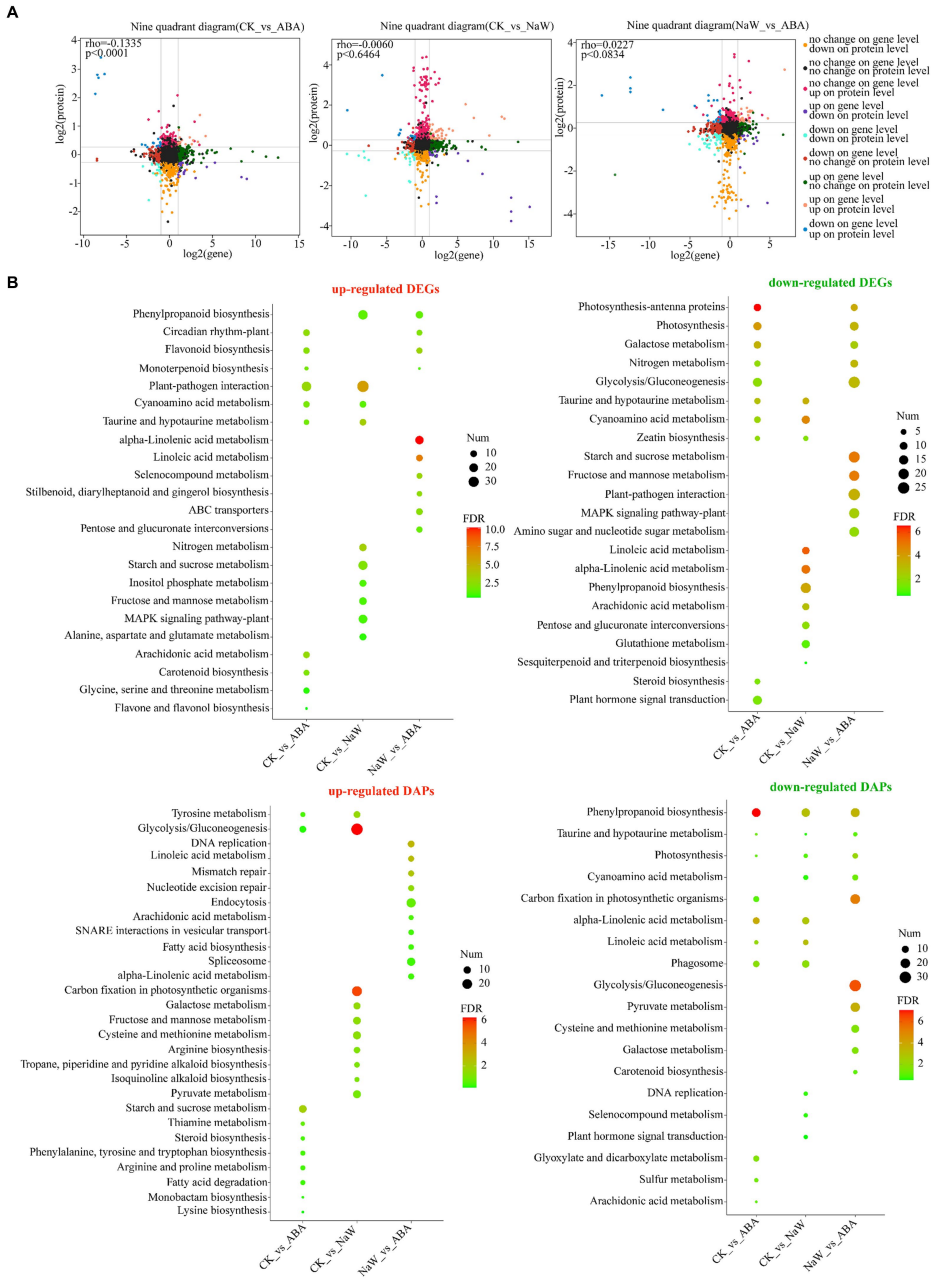
Expression correlation and functional annotation of DEGs and DAPs. **(A)** Correlation of DEG expression and DAP expression in nine quadrants in three groups. **(B)** KEGG enrichment analysis of up-and down-regulated DEGs and DAPs between three groups.

The proteome and transcriptome data integration showed that 74, 115 and 126 DAPs were matched with their DEGs. Among these, 32 (16 up- and 16 downregulated), 82 (51 up- and 31 downregulated) and 78 DAPs (29 up- and 49 downregulated) showed the same tendency as DEGs in the CK_ABA, CK_NaW and NaW_ABA groups, respectively, with 42, 33, and 48 showing the opposite tendency as DEGs (Table 1; Supplementary Tables S6, S7). Pearson’s correlation tests revealed positive and negative correlations between fold changes in DAPs and DEGs, most prominently in the CK_ABA comparison (Rho = −0.1335, *p* < 0.0001). Correlations were weaker in CK_NaW and NaW_ABA (Figure 2A). They suggested that under certain conditions, changes in protein levels may not be entirely determined by changes in mRNA levels.

Furthermore, the functional annotation for both up-and down-regulated DEGs/DAPs in the CK_ABA, CK_NaW, and NaW_ABA groups are set to investigate using KEGG enrichment analysis (Supplementary Tables S8, S9) and GO enrichment analysis (Supplementary Tables S10, S11). The results indicated that many cell wall-, starch-, and plant hormone-related KEGG pathways that were significantly enriched in DEGs and DAPs, including “Phenylpropanoid biosynthesis,” “Starch and sucrose metabolism,” “Glycolysis/Gluconeogenesis,” “alpha-Linolenic acid metabolism,” “Linoleic acid metabolism,” “Carotenoid biosynthesis,” and “Plant hormone signal transduction.” Specifically, the enrichment of “Phenylpropanoid biosynthesis” in DEGs was primarily influenced by down-regulated DEGs in the CK_NaW group, along with some up-regulated DEGs in the CK_NaW and NaW_ABA groups. In contrast, for DAPs, the enrichment was mainly driven by the downregulated DAPs in the CK_ABA group, followed by the downregulated DAPs in the CK_NaW and NaW_ABA groups. This differential enrichment highlights the distinct regulatory mechanisms affecting gene expression and protein abundance in response to different treatments (Figure 2B). Interestingly, in the results of the enrichment analysis of GO terms, a large number of downregulated DAPs in the CK_ABA group were enriched in “stress response” and “response to stimulus” (Supplementary Figure S5). In summary, the analysis suggests a complex relationship between DEGs and DAPs, which may be influenced by potentially involving additional regulatory mechanisms beyond transcriptional control.

### 3.5 Mining of target functional clusters of ABA-specific regulation

**Figure 3:**
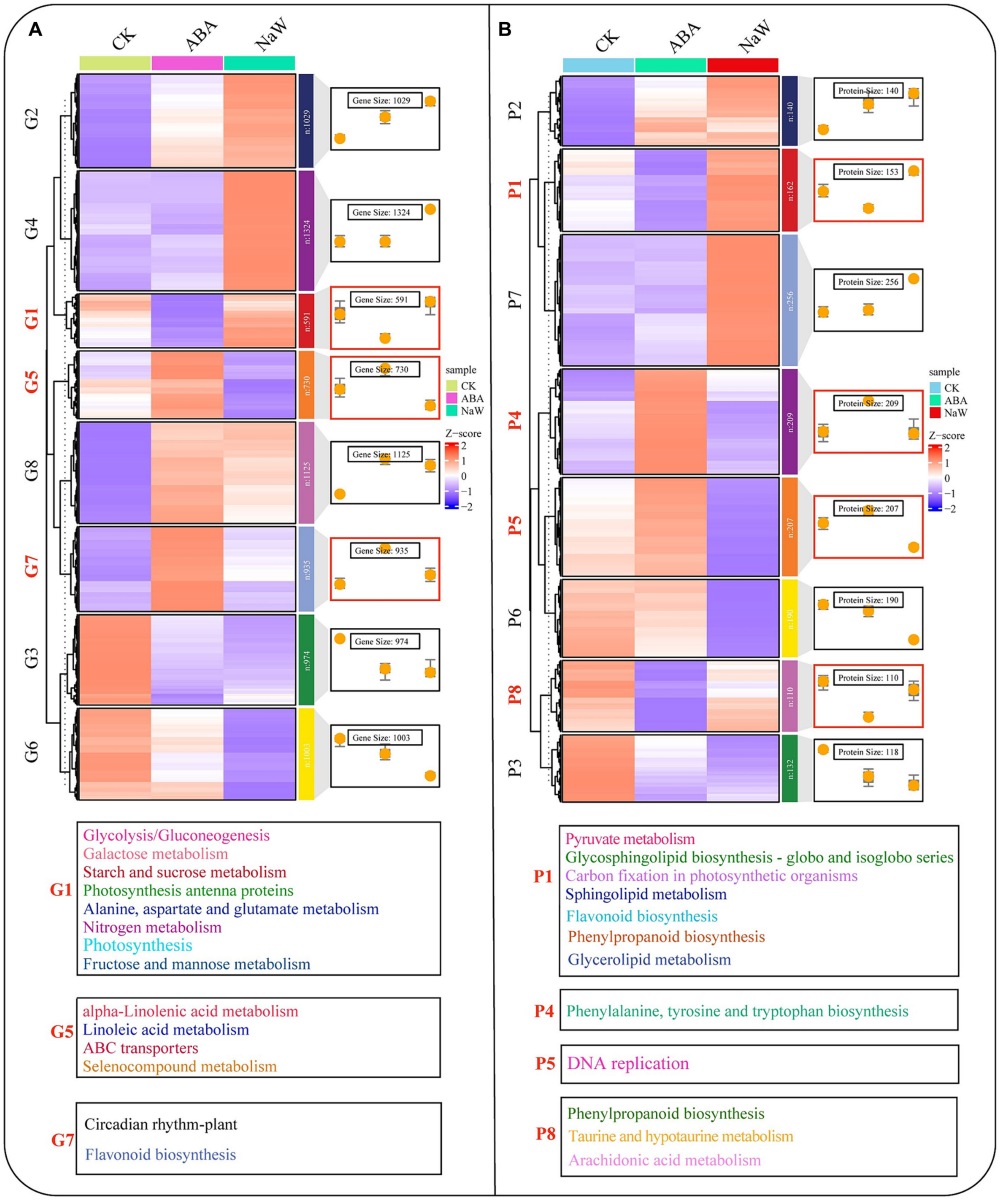
Cluster analysis of DEGs/DAPs and its functional enrichment. **(A)** Cluster analysis of DEG expression trend and its KEGG enrichment. **(B)** Cluster analysis of DAP expression trend and its GO enrichment.

To further explore the regulatory mechanisms by ABA, we utilized Fuzzy C-Means clustering to elucidate the underlying patterns within our identified DEGs and DAPs. Eight distinct clusters for DEGs (designated G1-G8) and DAPs (designated P1-P8; Supplementary Tables S12, S13). Cluster analysis revealed two major DEG and DAP groups: G5 (730), G7 (935), P4 (209), and P5 (207) were up-regulated, and G1 (591), P1 (153), and P8 (110) were down-regulated in response to ABA (Figure 3). The DEG clusters were analyzed using KEGG enrichment, and GO enrichment analysis was performed for DAP clusters. The results demonstrated that G1 was enriched in glycolysis gluconeogenesis, galactose metabolism, starch and sucrose metabolism. In contrast, G5 was enriched in alpha-linolenic acid metabolism, linoleic acid metabolism, ABC transporters, and selenocompound metabolism (Figure 3A; Supplementary Table S14). P1 was enriched in extracellular region, apoplast, alpha-galactosidase activity, and raffinose alpha-galactosidase activity, whereas P5 was enriched in sucrose synthase activity, S-adenosylmethionine biosynthetic process, MCM complex, methionine adenosyltransferase activity, lipid biosynthetic process, alcohol biosynthetic process, sucrose metabolic process, and DNA replication initiation; P4 was enriched in phenylalanine, tyrosine and tryptophan biosynthesis, whereas P8 was enriched in phenylpropanoid biosynthesis, taurine and hypotaurine metabolism, and arachidonic acid metabolism (Figure 3B; Supplementary Table S15). These findings suggest ABA exerts coordinated regulation of gene expression and protein abundance, specifically influencing pathways related to the cell wall (map00940/map00941/map02010), starch (map00500/map00010), and plant hormone (map00592/map00591) processes. Elucidating the precise mechanisms by which these clusters contribute to ABA-regulated processes will provide valuable insights into the molecular underpinnings of *P. heterophylla* tuberous root development.

### 3.6 Expressional regulation of DEGs and DAPs involved in tuberous root development

**Figure 4:**
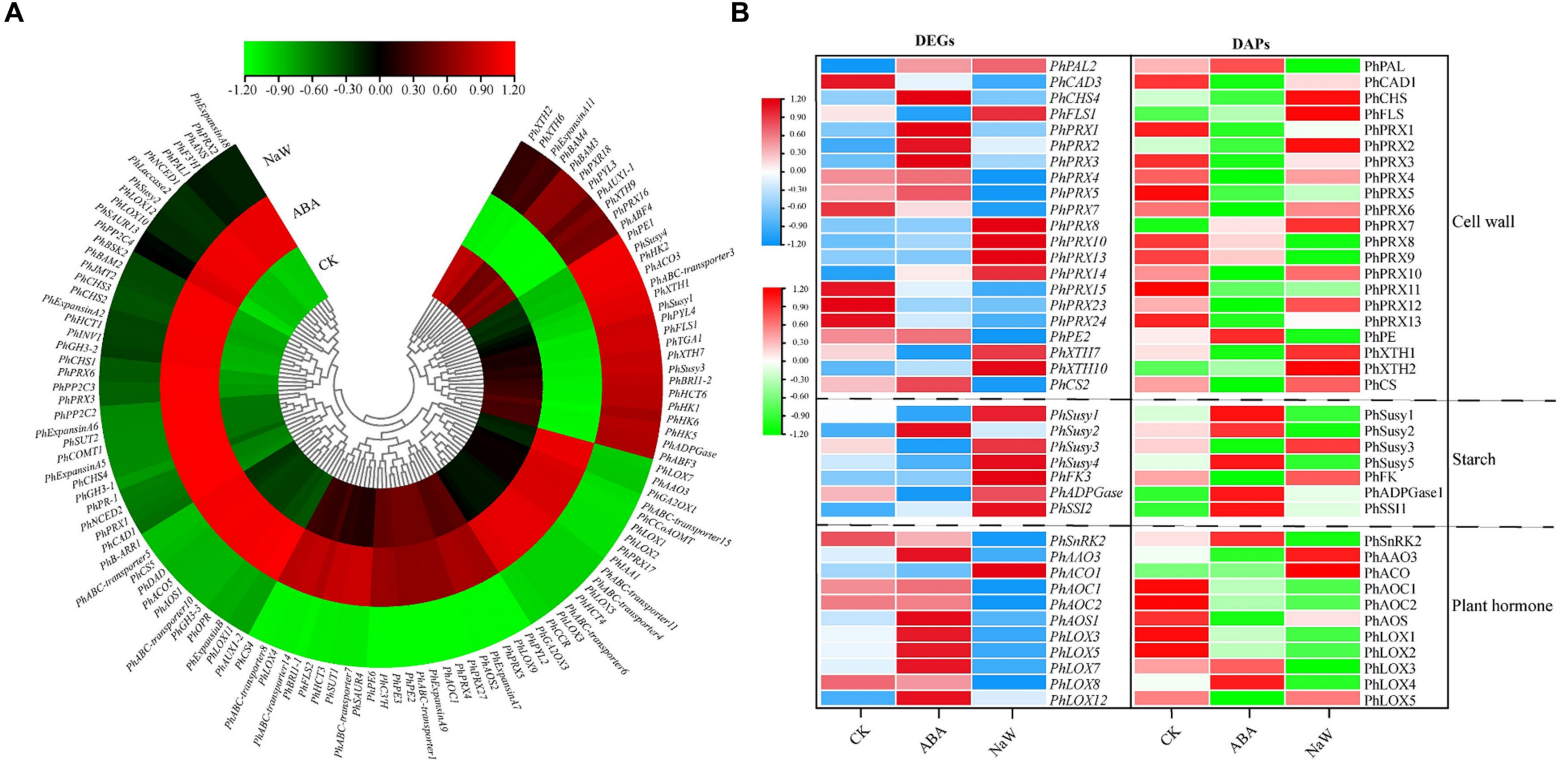
The DEGs/DAPs expression of structural genes in key pathways of *P. heterophylla* tuberous root development under exogenous ABA treatment. **(A)** Gene expression heat map of structural genes located in DEG clusters G1/G5/G7 in key pathways. **(B)** Gene and protein expression analysis of associated DEG-DAPs of structural genes in key pathways.

A total of 242 DEGs were identified across three critical pathways: cell wall formation, starch metabolism, and plant hormone signaling/biosynthesis (Supplementary Table S16). Specifically, ABA significantly up-regulates the expression of several genes, including *PhAAO3,* involved in ABA biosynthesis; *PhGA2OX1/3* involved in gibberellin biosynthesis, *PhLOX1/2/3/5/7* involved in jasmonic acid biosynthesis, *PhIAA1* involved in auxin signal transduction, and *PhCCoAOMT*, *PhPRX17*, *PhABC-transporter4/6/11/15*, *PhHCT4*, *PhCCR* involved in secondary cell wall formation. Conversely, ABA significantly down-regulates the expression of some genes, including *PhPE1* involved in primary cell wall formation, *PhSusy1/3/4*, *PhHK1/2/5/6*, *PhADPGase* involved in starch synthesis, *PhXTH1/7* involved in cell wall expansion, *PhPYL4*, *PhABF3* involved in ABA signal transduction, *PhFLS1*, *PhABC-transporter3*, *PhHCT6* involved in secondary cell wall biosynthesis, *PhTGA1* involved in salicylic acid signal transduction, and *PhBRI1-2* involved in brassinosteroid signal transduction, *PhACO3* involved in ethylene biosynthesis (Figure 4A). The ABA-specific up-regulation or down-regulation genes possibly act as a regulatory mechanism for balancing growth and tuberous root development in *P. heterophylla*.

There were 39 DEG-DAPs related to cell wall formation, starch metabolism, and plant hormone signaling/biosynthesis was identified (Figure 4B; Supplementary Figures S6–S8). Lignin synthesis involves a series of enzymes, among which peroxidases (PRX) play a catalytic role in the polymerization of lignin monomers (39). In this study, PhPRX5 showed down-regulated expression at the transcriptional and protein levels in the CK_vs_NaW group. Pectinase (PE), catalyzing the hydrolysis of pectin, contributes to the remodeling of the cell wall, enabling plants to undergo new growth and transformations (40). PhPE showed up-regulated expression at both the transcriptional and protein levels in the ABA_vs_NaW group. The xyloglucan endotransglucosylase/hydrolase (XET/XEH, also named XTH) family is a multigene family, the function of which plays a significant role in cell-wall rebuilding and stress tolerance in plants (41). PhXTH1 showed down-regulated expression at both the transcriptional and protein levels in the ABA_vs_NaW group. Sucrose synthase (Susy) can catalyze the reversible cleavage of sucrose into fructose and uridine diphosphate glucose (UDP-G), thereby providing precursor molecules for starch synthesis (42). PhSusy3 showed down-regulated expression at both the transcriptional and protein levels in the CK_vs_ABA group. In our research, jasmonic acid may be one of the essential hormones involved in regulating *P. heterophylla* tuberous root development by ABA. PhAOC1 showed down-regulated expression at the transcriptional and protein levels in the CK_vs_NaW group. PhLOX3 showed up-regulated expression in the transcriptional and protein levels in the ABA_vs_NaW group (Table 2). These DEG or DAPs may play a crucial role in ABA’s regulation of *P. heterophylla* tuberous root development.

**Table 2:** DEG-DAPs expression correlation information of pathway structure genes.

Gene_id(name)	Protein_id(name)	pathway	DEG_cluster	DAP_cluster	Expression correlation
TRINITY_DN18158_c2_g2(*PhCAD3*)	TRINITY_DN18158_c2_g2_i7_m.423581(PhCAD1)	cell wall	G6	P8	——
TRINITY_DN16285_c1_g1(*PhPAL2*)	TRINITY_DN16285_c1_g1_i2_m.19561(PhPAL)	cell wall	G8	P5	——
TRINITY_DN10247_c2_g2(*PhPRX1*)	TRINITY_DN10247_c2_g2_i1_m.94858(PhPRX1)	cell wall	G7	P3	CK_vs_ABA_DEG up DAP down
TRINITY_DN10247_c2_g6(*PhPRX2*)	TRINITY_DN10247_c2_g6_i2_m.94881(PhPRX2)	cell wall	G7	P1	——
TRINITY_DN10367_c6_g2(*PhPRX3*)	TRINITY_DN10367_c6_g2_i2_m.429931(PhPRX3)	cell wall	G7	P8	CK_vs_ABA_DEG up DAP down
TRINITY_DN10367_c7_g6(*PhPRX4*)	TRINITY_DN10367_c7_g6_i1_m.429944(PhPRX4)	cell wall	G5	P8	ABA_vs_NaW_DEG up DAP down
TRINITY_DN10453_c1_g1(*PhPRX5*)	TRINITY_DN10453_c1_g1_i1_m.1623(PhPRX5)	cell wall	G5	P3	CK_vs_NaW_DEG down DAP down
TRINITY_DN10551_c3_g2(*PhPRX7*)	TRINITY_DN10551_c3_g2_i3_m.176623(PhPRX6)	cell wall	G6	P8	——
TRINITY_DN10572_c0_g1(*PhPRX8*)	TRINITY_DN10572_c0_g1_i1_m.175326(PhPRX7)	cell wall	G4	P2	CK_vs_NaW_DEG up DAP up
TRINITY_DN10572_c2_g1(*PhPRX10*)	TRINITY_DN10572_c2_g1_i2_m.175334(PhPRX8)	cell wall	G4	P6	CK_vs_NaW_DEG up DAP down
TRINITY_DN11441_c0_g4(*PhPRX13*)	TRINITY_DN11441_c0_g4_i1_m.207802(PhPRX9)	cell wall	G4	P6	CK_vs_NaW_DEG up DAP down
TRINITY_DN11537_c0_g5(*PhPRX14*)	TRINITY_DN11537_c0_g5_i1_m.136318(PhPRX10)	cell wall	G2	P8	ABA_vs_NaW_DEG down DAP down, CK_vs_ABA_DEG up DAP down
TRINITY_DN11754_c1_g1(*PhPRX15*)	TRINITY_DN11754_c1_g1_i2_m.287462(PhPRX11)	cell wall	G6	P3	CK_vs_NaW_DEG down DAP down
TRINITY_DN17798_c0_g1(*PhPRX23*)	TRINITY_DN17798_c0_g1_i3_m.459779(PhPRX12)	cell wall	G3	P8	CK_vs_ABA__DEG down DAP down
TRINITY_DN18092_c1_g1(*PhPRX24*)	TRINITY_DN18092_c1_g1_i3_m.24007(PhPRX13)	cell wall	G3	P8	CK_vs_NaW_DEG down DAP down
TRINITY_DN15089_c2_g4(*PhCHS4*)	TRINITY_DN15089_c2_g4_i1_m.108148(PhCHS)	cell wall	G7	P1	ABA_vs_NaW_DEG up DAP down
TRINITY_DN16221_c0_g1(*PhFLS1*)	TRINITY_DN16221_c0_g1_i1_m.19878(PhFLS)	cell wall	G1	P7	ABA_vs_NaW_DEG down DAP down, CK_vs_ABA_DEG down DAP up
TRINITY_DN15182_c3_g2(*PhPE2*)	TRINITY_DN15182_c3_g2_i1_m.276189(PhPE)	cell wall	G5	P5	ABA_vs_NaW_DEG up DAP up
TRINITY_DN15700_c4_g1(*PhXTH7*)	TRINITY_DN15700_c4_g1_i1_m.69573(PhXTH1)	cell wall	G1	P1	ABA_vs_NaW_DEG down DAP down
TRINITY_DN18842_c4_g1(*PhXTH10*)	TRINITY_DN18842_c4_g1_i2_m.311198(PhXTH2)	cell wall	G4	P7	CK_vs_NaW_DEG up DAP up, ABA_vs_NaW_DEG down DAP down
TRINITY_DN13969_c2_g2(*PhCS4*)	TRINITY_DN13969_c2_g2_i1_m.386260(PhCS)	cell wall	G5	P8	ABA_vs_NaW_DEG up DAP down
TRINITY_DN11518_c0_g3(*PhADPGase*)	TRINITY_DN11518_c0_g3_i1_m.137000(PhADPGase1)	starch	G1	P4	——
TRINITY_DN18110_c2_g1(*PhFK3*)	TRINITY_DN18110_c2_g1_i1_m.426134(PhFK)	starch	G4	P8	ABA_vs_NaW_DEG down DAP down
TRINITY_DN17637_c0_g1(*PhSSI2*)	TRINITY_DN17637_c0_g1_i1_m.117613(PhSSI1)	starch	G2	P4	——
TRINITY_DN11765_c2_g1(*PhSusy1*)	TRINITY_DN11765_c2_g1_i1_m.290159(PhSusy1)	starch	G1	P5	ABA_vs_NaW_DEG down DAP up
TRINITY_DN12800_c0_g1(*PhSusy2*)	TRINITY_DN12800_c0_g1_i3_m.437823(PhSusy2)	starch	G7	P5	CK_vs_NaW_DEG up DAP down
TRINITY_DN13032_c1_g1(*PhSusy3*)	TRINITY_DN13032_c1_g1_i4_m.294032(PhSusy3)	starch	G1	P8	CK_vs_ABA__DEG down DAP down
	TRINITY_DN13032_c1_g1_i5_m.294045(PhSusy4)			P1	——
TRINITY_DN9947_c1_g2(*PhSusy4*)	TRINITY_DN9947_c1_g2_i2_m.121314(PhSusy5)	starch	G1	P5	ABA_vs_NaW_DEG down DAP up
TRINITY_DN16489_c0_g5(*PhAAO3*)	TRINITY_DN16489_c0_g5_i1_m.115819(PhAAO3)	plant hormone	G5	P1	ABA_vs_NaW_DEG up DAP down
TRINITY_DN17406_c2_g1(*PhSnRK2*)	TRINITY_DN17406_c2_g1_i1_m.379082(PhSnRK2)	plant hormone	G6	P5	CK_vs_NaW_DEG down DAP down
TRINITY_DN12672_c2_g2(*PhACO1*)	TRINITY_DN12672_c2_g2_i2_m.75444(PhACO)	plant hormone	G4	P7	CK_vs_NaW_DEG up DAP up, ABA_vs_NaW_DEG down DAP down
TRINITY_DN15210_c3_g1(*PhAOC1*)	TRINITY_DN15210_c3_g1_i5_m.300424(PhAOC1)	plant hormone	G5	P6	CK_vs_NaW_DEG down DAP down, ABA_vs_NaW_DEG up DAP up
TRINITY_DN13828_c1_g1(*PhAOC2*)	TRINITY_DN13828_c1_g1_i13_m.150776(PhAOC2)	plant hormone	G6	P3	CK_vs_NaW_DEG down DAP down
TRINITY_DN13310_c0_g2(*PhAOS1*)	TRINITY_DN13310_c0_g2_i1_m.392513(PhAOS)	plant hormone	G5	P8	——
TRINITY_DN10349_c2_g1(*PhLOX3*)	TRINITY_DN10349_c2_g1_i8_m.433148(PhLOX1)	plant hormone	G5	P6	CK_vs_NaW_DEG down DAP down
TRINITY_DN14950_c1_g1(*PhLOX5*)	TRINITY_DN14950_c1_g1_i4_m.157306(PhLOX2)	plant hormone	G5	P6	CK_vs_NaW_DEG down DAP down, ABA_vs_NaW_DEG up DAP up
TRINITY_DN12956_c1_g1(*PhLOX7*)	TRINITY_DN12956_c1_g1_i1_m.79217(PhLOX3)	plant hormone	G5	P5	ABA_vs_NaW_DEG up DAP up
TRINITY_DN12956_c1_g3(*PhLOX8*)	TRINITY_DN12956_c1_g3_i1_m.79225(PhLOX4)	plant hormone	G6	P5	CK_vs_NaW_DEG down DAP down
TRINITY_DN17848_c3_g1(*PhLOX12*)	TRINITY_DN17848_c3_g1_i3_m.449700(PhLOX5)	plant hormone	G7	P8	——

### 3.7 Analysis of ABA-regulated potential TFs affecting tuberous root development

**Figure 5:**
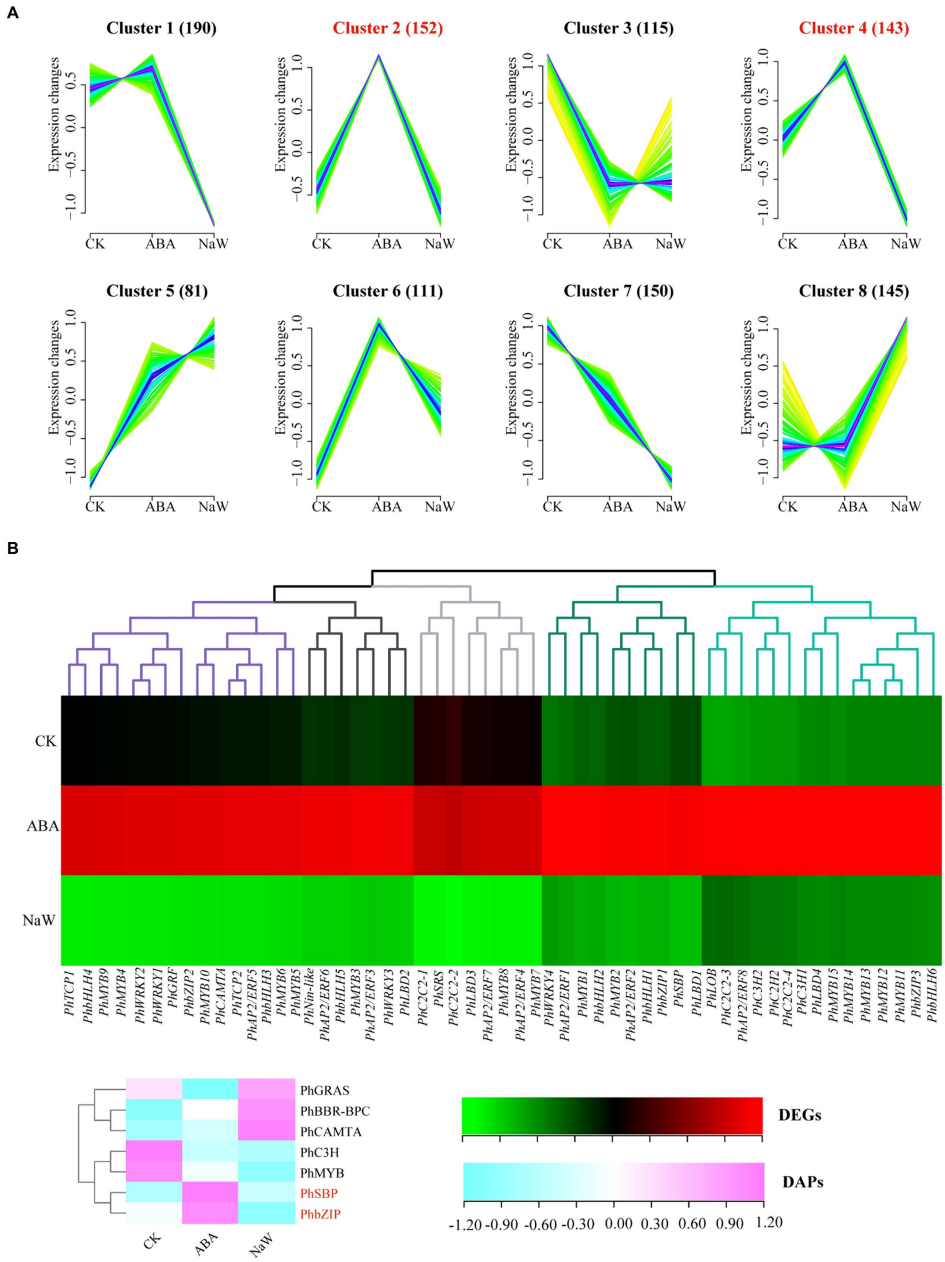
Analysis of ABA-regulated TFs involved in *P. heterophylla* tuberous root development. **(A)** Cluster analysis of TF expression trend. **(B)** Gene expression heat map of TF DEGs in cluster 2/4 and protein expression heat map of TF DAPs.

Transcriptomic analysis of ABA-treated *P. heterophylla* tuberous roots identified 1,087 TFs spanning 38 families. Utilizing the fuzzy c-means clustering algorithm of Mfuzz, these TFs were categorized into 8 clusters (Figure 5A; Supplementary Table S17). The expression patterns of TFs within Cluster 2 and Cluster 4 align with those of the structural genes in DEG clusters G5 and G7, exhibiting a contrasting trend to the expression pattern observed in G1. Within these two clusters, we identified 55 significantly differentially expressed TFs, which we designated core TFs (Supplementary Table S18). Additionally, we predicted 27 TF proteins in the proteomic data, 7 of which showed differential expression (Supplementary Table S19). Using a heatmap, we further visualized the expression patterns of these 55 differentially expressed TF genes and 7 differentially expressed TF proteins under ABA treatment (Figure 5B). Co-expressed genes may have similar functions in biological processes or participate in the same biological pathways. Notably, in our results, *PhMYB4*, *PhMYB5*, *PhMYB6*, *PhMYB9*, *PhMYB10*, *PhbHLH3*, *PhbHLH4*, *PhTCP1*, *PhTCP2*, *PhWRKY1*, *PhWRKY2*, *PhGRF*, *PhbZIP2*, *PhCAMTA*, and *PhAP2/ERF5* show extremely similar expression patterns to pathway structure DEGs. Furthermore, we found that ABA up-regulated two TF DAPs (PhbZIP, PhSBP) at the protein level.

**Figure 6:**
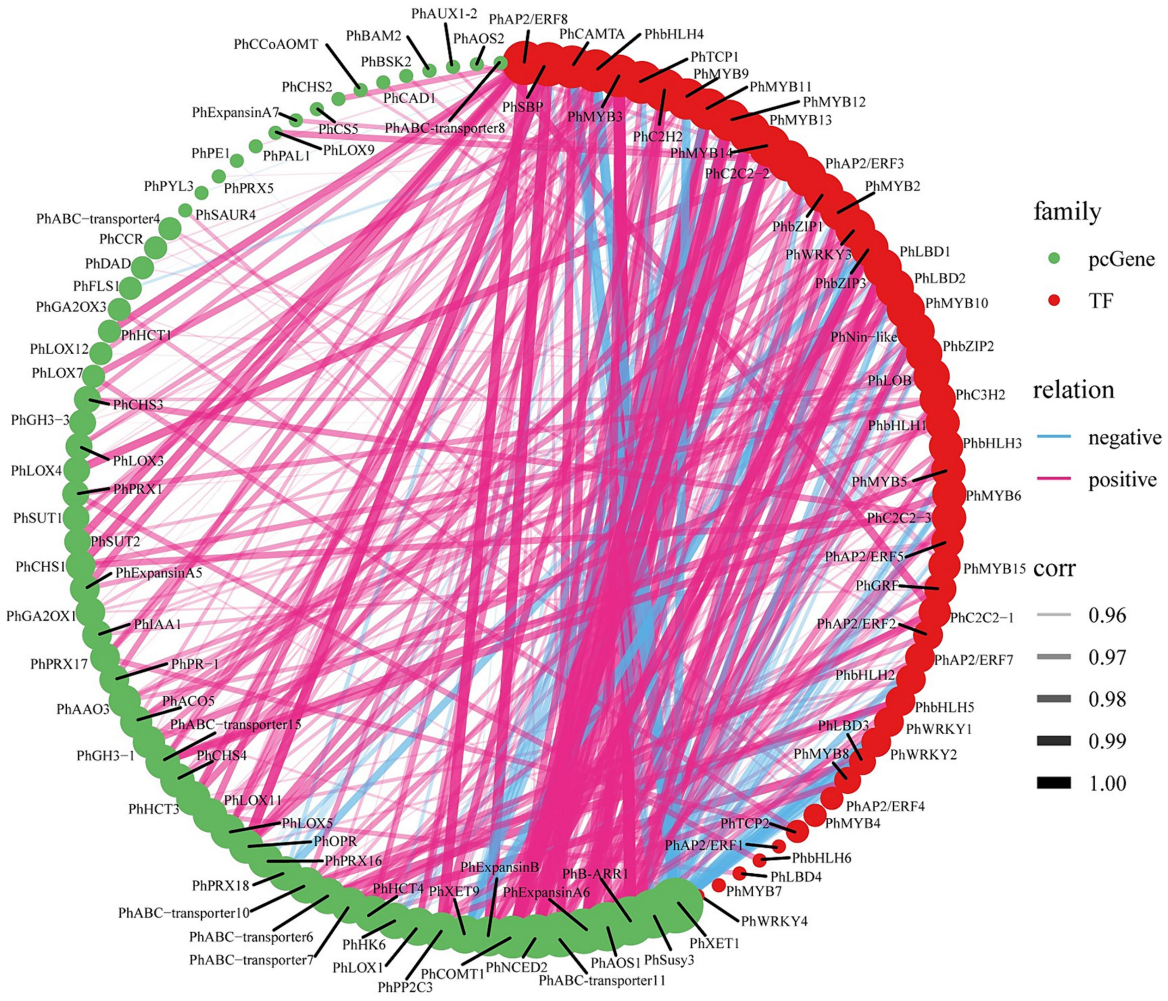
ABA-regulated gene-TF co-expression analysis. The size of the circles represents the number of associations or interactions, with larger circles indicating a higher number of associations with other genes within the network. Significant correlations for all filtered genes and TFs (abs corr>0.95 and *p*-value<0.05).

We calculated the co-expression relationships of all screened pathway structure genes and TFs at the mRNA level. To determine the significance of these relationships, we set a threshold (abs corr >0.95 and *p*-value <0.05). We presented the results of these significant correlations as a network diagram (Figure 6; Supplementary Table S20). In this network diagram, the nodes represent genes or TFs, and the edges represent their correlations. Co-expression network analysis has revealed an important transcriptional regulatory network. In this network, *PhMYB10* and *PhbZIP2* interact to positively regulate *PhABC-transporter7*, *PhIAA1*, *PhAAO3*, *PhB-ARR1*, and *PhAOS1*, and negatively regulate *PhSusy3* and *PhXTH1*.

### 3.8 Identification of regulatory network mediating ABA regulation of tuberous root development

**Figure 7:**
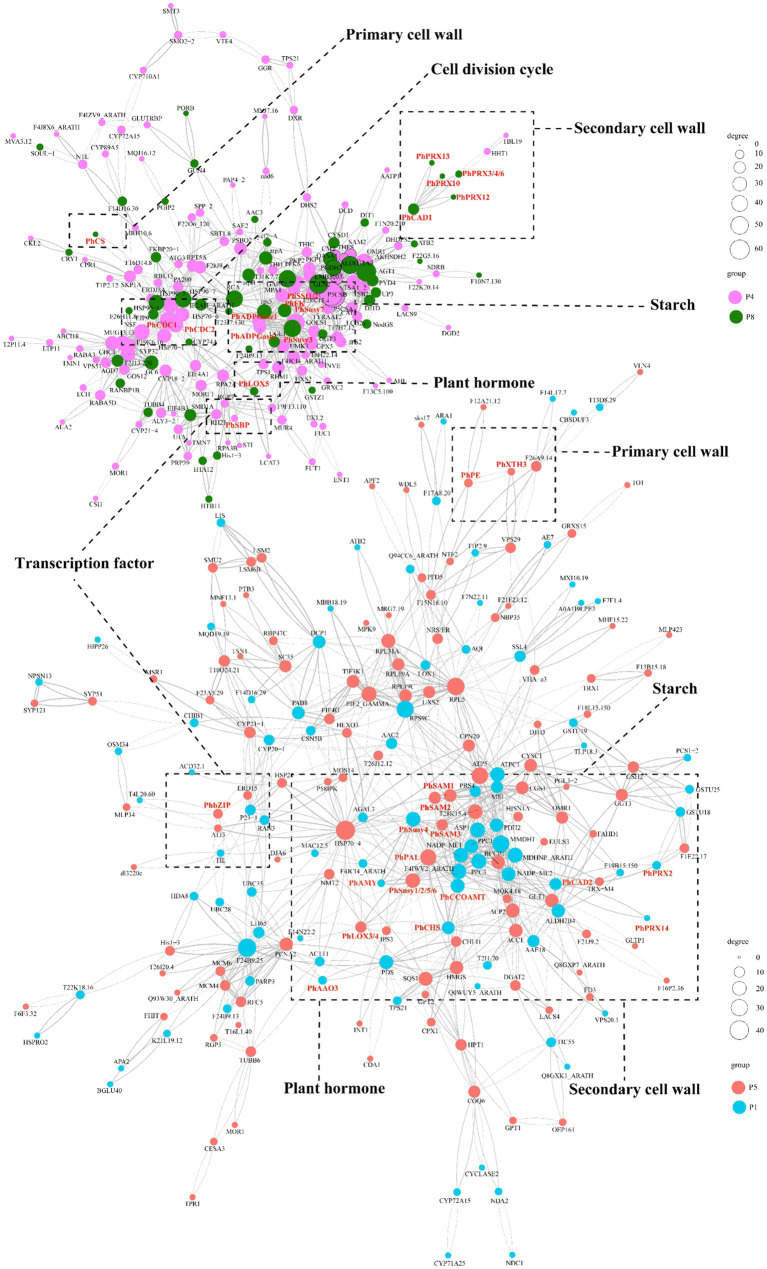
PPI prediction of the functional network in ABA-regulated *P. heterophylla* tuberous root development. DAP conducted network 1 in clusters P4/P8, and DAP conducted network 2 in clusters P1/P5.

At the protein level, we conducted protein interaction prediction studies on the DAPs in clusters P4/P8 (Supplementary Table S21) and clusters P1/P5 (Supplementary Table S22). We have constructed a network diagram for the proteins with the highest interaction rankings (Figure 7; Supplementary Table S23). The results show that the ABA-specifically up-regulated TF PhbZIP interacts with ABA-specifically downregulated pathway structural proteins (PhAAO3, PhCHS, PhCCoAOMT, PhCAD2, PhSusy3, PhXTH1, PhPRX2, PhPRX14), as well as with ABA-specifically up-regulated proteins (PhLOX3, PhLOX4, PhSAM1, PhSAM2, PhSAM3, PhPAL, PhSusy1, PhSusy2, PhSusy5, PhSusy6, PhPE, PhXTH3). Additionally, the ABA-upregulated TF PhSBP interacts with ABA-upregulated pathway structural proteins (PhCDC1, PhCDC2, PhSusy7, PhSSI1, PhSSI2, PhADPGase1, PhADPGase2) and with ABA-downregulated pathway structural proteins (PhLOX5, PhSusy4, PhFK, PhPRX3, PhPRX4, PhPRX6, PhPRX10, PhPRX12, PhPRX13, PhCAD1, PhCS). In particular, proteins positively regulated by PhbZIP and PhSBP are crucial in developing tuberous roots in *P. heterophylla*. These proteins are vital to an ABA-regulated network, influencing cell wall composition, starch synthesis, hormone signaling, and cell division processes.

## 4 Discussion

The phytohormone ABA is a critical factor that can balance endogenous plant hormones and regulate plant growth and metabolism (7). Previous studies have shown that ABA can promote primary root growth by accelerating root cell division, increasing the size and number of meristem cells, and promoting cell wall alkalinization and elongation (8). Similar results were found in this study, which reduced cell division and promoted expansion, as well as starch granule accumulation, leading to an increase in the diameter and a decrease in the length of *P. heterophylla* tuberous roots.

The tuberous root development is a complex and delicate process involving the expression and regulation of various enzymes and genes. ABA and GA, as antagonistic hormones, play an essential role in regulating the root development (43). Increasing accumulation of IAA and ABA and reduction in GA promote Chinese cabbage swollen root formation (43). Overexpression of the gibberellin degradation gene *StGA2ox1* in potatoes promotes tuberization, and silencing *StGA2ox1* delayed tuberization (44). These results indicated that gibberellin played a negative regulatory role in tuberization. In this study, ABA treatment up-regulated the ABA biosynthesis gene *PhAAO3* and gibberellin degradation gene *PhGA2OX1/3,* promoting tuberous roots’ development. This finding is consistent with previous study results, emphasizing the importance of maintaining a balance between ABA and GA during the development of plant tuberous roots (43, 45, 46).

Additionally, ABA may play a role in regulating the response of *P. heterophylla* to stress and defense mechanisms by up-regulating the biosynthesis genes of jasmonic acid (*PhLOX1/2/3/5/7*), thereby affecting the growth of tuberous roots and the accumulation of secondary metabolites in *P. heterophylla* (47, 48). ABA often promotes root growth by increasing the action of auxin. By up-regulating *PhIAA1* involved in auxin signaling, ABA may enhance the role of this auxin in developing the tuberous roots of *P. heterophylla* (49). The regulation of ABA on the formation of secondary cell walls (*PhCCoAOMT*, *PhPRX17*, *PhABC-transporter3/4/6/11/15*, *PhHCT4/6*, *PhCCR*, *PhFLS1*), the relaxation/expansion of primary cell walls (*PhPE1*, *PhXTH1/7*), and starch metabolism (*PhSusy1/3/4*, *PhHK1/2/5/6*, *PhADPGase*) reveal the critical role of ABA in regulating cell expansion and starch accumulation of tuberous root. Further research found that ABA explicitly regulated four candidate proteins (PhPE, PhXTH1, PhSusy3, and PhLOX3) at transcription and protein levels. The current study suggests that these 4 candidate proteins may play a significant role in ABA regulation in developing *P. heterophylla* tuberous root.

TFs are regulatory proteins that activate or inhibit gene transcription by binding to specific promoter sequences in most instances (50). MYB is one of the families of transcriptional regulators with a large number of members in plants. Previous studies have shown that MYB proteins are involved in the regulation of multiple processes, such as root development (51), cell division (52), secondary cell wall development (53), and secondary metabolite synthesis (54). In addition, the MYB also has been validated to be involved in the metabolic pathways of GA (55), ABA (56), auxin (51), cytokinin (57), and jasmonic acid (58). It will be seen from this that a complex network of synergistic or antagonistic interactions among various hormones regulates vital processes in *P. heterophylla* tuberous root, and 5 *PhMYBs* appear to mediate this crucial process. Our transcriptional regulatory correlation network analysis of TFs and structural genes suggests that under exogenous ABA treatment, *PhMYB10* may interact with *PhbZIP2* to mediate auxin signaling (*PhIAA1*), ABA biosynthesis (*PhAAO3*), cytokinin signaling (*PhB-ARR1*), and jasmonic acid biosynthesis (*PhAOS1*), significantly promoting the expression of *PhABC-transporter7*, may enhance the transmembrane transport of lignin monomers, which is essential for the formation of lignin in the secondary cell wall (59); and significantly inhibiting the expression of *PhSusy3*, may reduce the synthesis of starch granule; and significantly inhibiting the expression of *PhXTH1*, may reduce the relaxation of the cell wall (60). Under stress conditions, bZIP proteins participate in the ABA response, interacting with corresponding ABRE-binding factors (ABFs) or ABRE-binding proteins (AREBs) to regulate the transcription of downstream target genes (13, 61, 62). Thus, the transcriptional regulatory mechanisms mediated by *PhMYB10* and *PhbZIP2*, may play a crucial role in the ABA-regulated network of tuberous root development in *P. heterophylla*.

The ABA signaling is precisely regulated by numerous post-translational factors in fluctuating environments (7). In this study, we found that two important TF proteins (PhbZIP and PhSBP) were strongly induced by ABA treatment. However, the expression of their corresponding transcripts did not show significant changes under ABA treatment, perhaps due to differences in the stability of protein/mRNA or the effect of miRNAs in post-transcriptional regulation (63). Protein interactions are crucial for understanding cellular functions and biological mechanisms (64). Through protein interaction network analysis, 19 candidate interacting proteins were identified, including three PhSAMs (PhSAM1, PhSAM2, PhSAM3), which are key enzymes in the biosynthesis of ethylene, converting the direct precursor S-adenosylmethionine (SAM) into the ethylene precursor 1-aminocyclopropane-1-carboxylic acid (ACC), and may play an important role in tuberous root development (65, 66). Similarly, based on previous research, we have predicted more regulatory mechanisms that affect the development of tuberous roots in *P. heterophylla*: ABA may mediate the regulation of PhPAL by PhbZIP, where PhPAL is encoded by phenylalanine ammonia-lyase (PAL) and catalyzes the conversion of phenylalanine to cinnamic acid, the first step in lignin biosynthesis, thereby promoting the formation of secondary cell walls (67); ABA may also mediate the regulation of two PhCDCs (PhCDC1, PhCDC2) by PhSBP, which are encoded by cell division control proteins (CDC), thus promoting different stages of the cell cycle (34, 68); ABA could further mediate the regulation of two PhSSIs (PhSSI1, PhSSI2) by PhSBP, which are encoded by starch synthase I (SSI) and are involved in and promote starch synthesis (69); finally, ABA can regulate two PhADPGases (PhADPGase1, PhADPGase2) through PhSBP, which are encoded by glucose-1-phosphate adenylyltransferase (ADPGase), providing the substrate ADP-glucose for starch synthesis and directly promoting the rate of starch synthesis (70). In addition, five PhSusys (PhSusy1, PhSusy2, PhSusy5, PhSusy6), PhPE, PhXTH3, and two PhLOXs (PhLOX3, PhLOX4) could be mediated by PhbZIP, while PhSusy7 could be mediated by PhSBP. It follows that the network of regulatory mechanisms mediated by PhbZIP and PhSBP may be very important for the role of ABA in promoting the development of tuberous roots in *P. heterophylla*.

**Figure 8:**
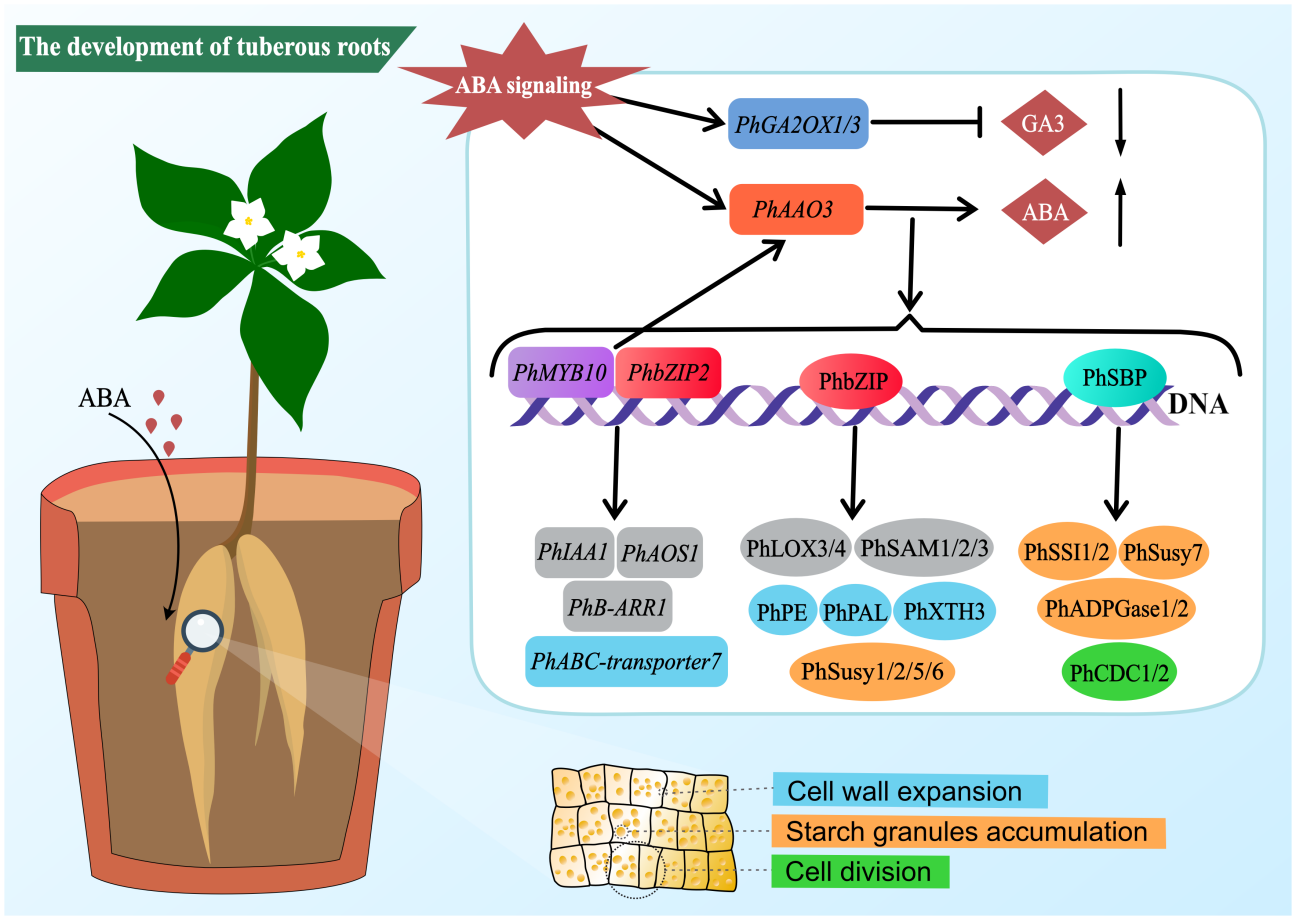
Schematic diagram of ABA-mediated regulatory network in tuberous root development of *P. heterophylla*.

In summary, this study elucidates that ABA can promote the development of *P. heterophylla* tuberous roots by regulating cell division and expansion and accumulating starch granules. Transcriptome and proteome analyses reveal a complex molecular network of ABA regulation in developing *P. heterophylla* tuberous roots, including critical pathways related to cell walls, starch metabolism, and plant hormone signal transduction/biosynthesis. In particular, we identified a series of TFs (such as PhMYB10, PhbZIP2, and PhSBP) closely related to ABA signal transduction and constructed their co-expression network with pathway structural genes. Moreover, our research shows that ABA treatment can precisely regulate the expression of these TFs (Figure 8). These findings provide an important molecular basis for further research on the role of ABA in developing *P. heterophylla* tuberous roots and offer new strategies for enhancing its medicinal value.

## Data availability statement

The datasets presented in this study can be found in online repositories. The names of the repository/repositories and accession number(s) can be found in the article/Supplementary material.

## Author contributions

CW: Data curation, Formal analysis, Investigation, Methodology, Writing – original draft. JY: Formal analysis, Investigation, Methodology, Writing – review & editing. QP: Data curation, Formal analysis, Investigation, Writing – review & editing. PZ: Conceptualization, Formal analysis, Investigation, Methodology, Writing – review & editing. JL: Conceptualization, Data curation, Funding acquisition, Investigation, Methodology, Project administration, Writing – review & editing.

## Footnotes

^1^ https://github.com/jstjohn/SeqPrep

^2^ https://github.com/najoshi/sickle

^3^ http://geneontology.org/

^4^ http://www.genome.jp/kegg/
