# Supplementary material for: Integrated transcriptomic and proteomic analysis of exogenous abscisic acid regulation on tuberous root development in Pseudostellaria heterophylla
Source: Front Nutr. 2024 Jul 5;11:1417526. doi: 10.3389/fnut.2024.1417526 (PMC11258014; doi:10.3389/fnut.2024.1417526)
Supplement: Supplementary file 1 [file Image_1.pdf]

Supplementary Material

1 Supplementary Figures

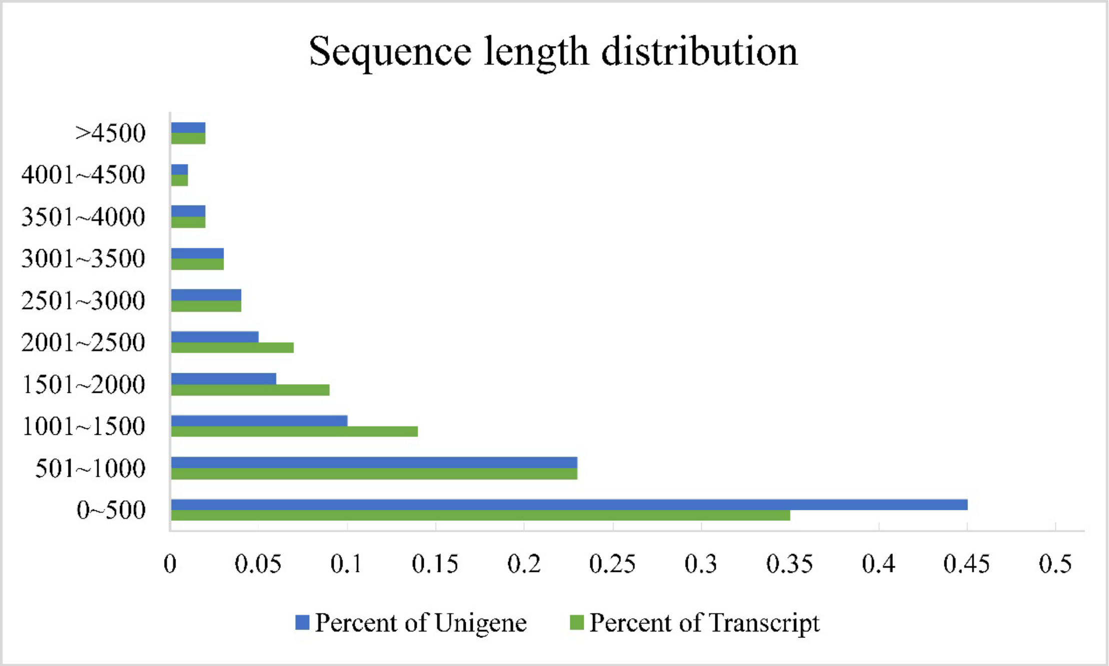

Supplementary Figure S1. Sequence length distribution.

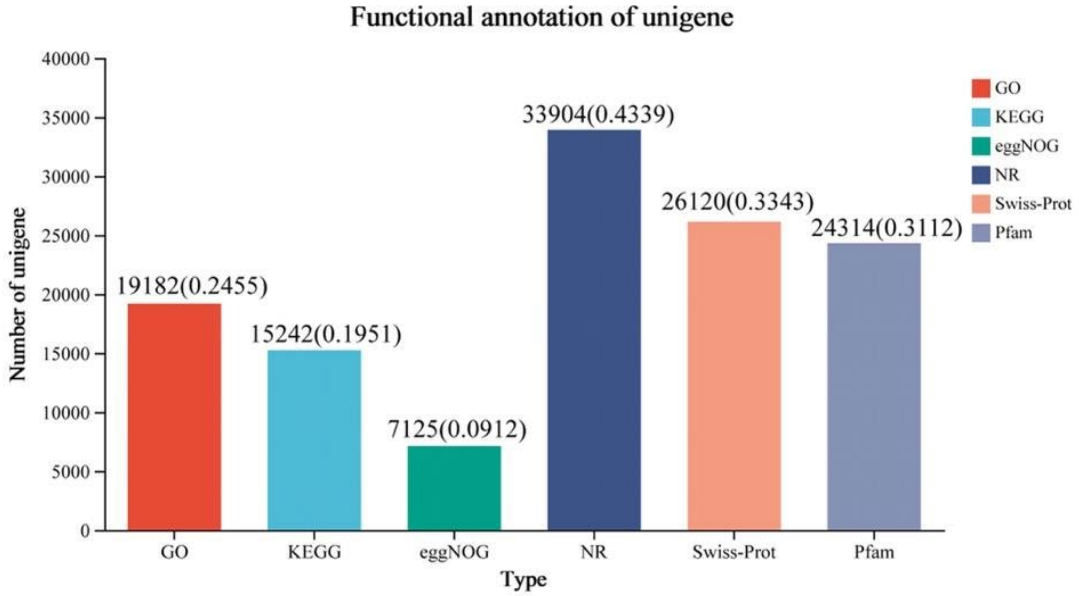

Supplementary Figure S2. Statistics of functional annotation of unigenes.

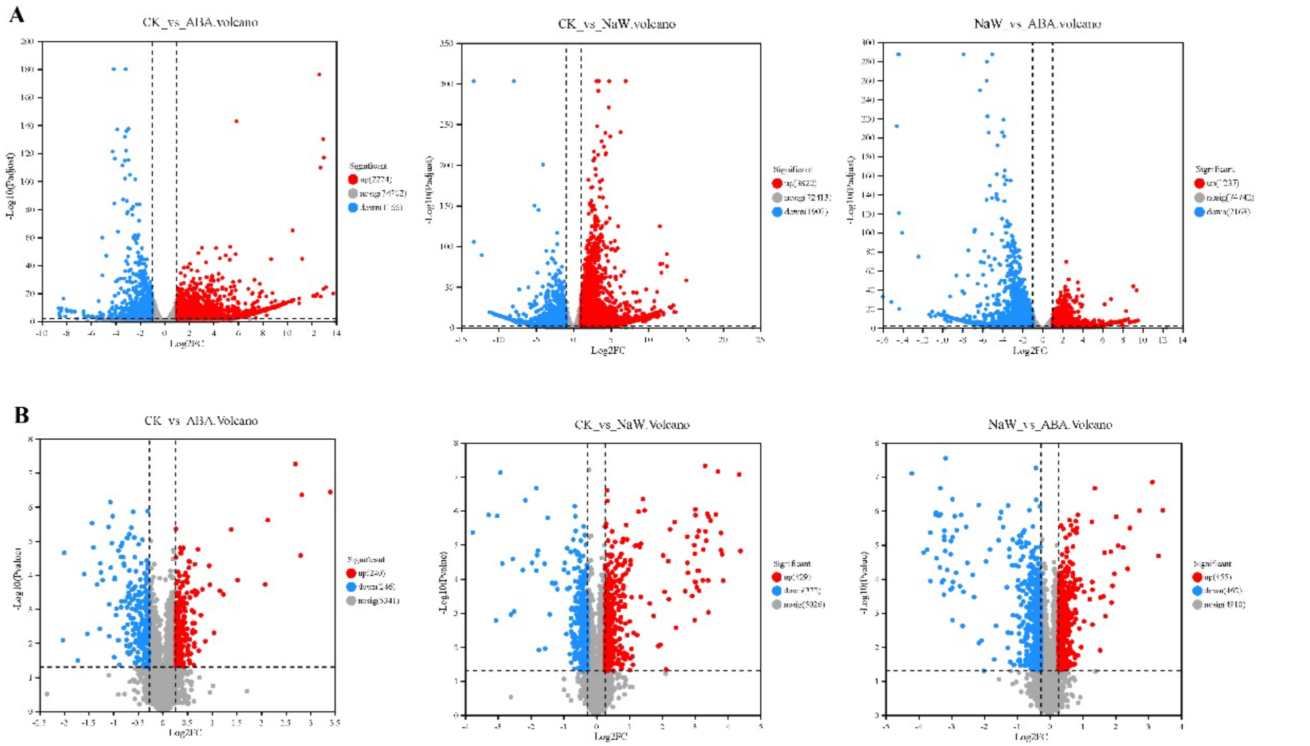

Supplementary Figure S3. Differential gene (A) and protein (B) expression volcano plot.

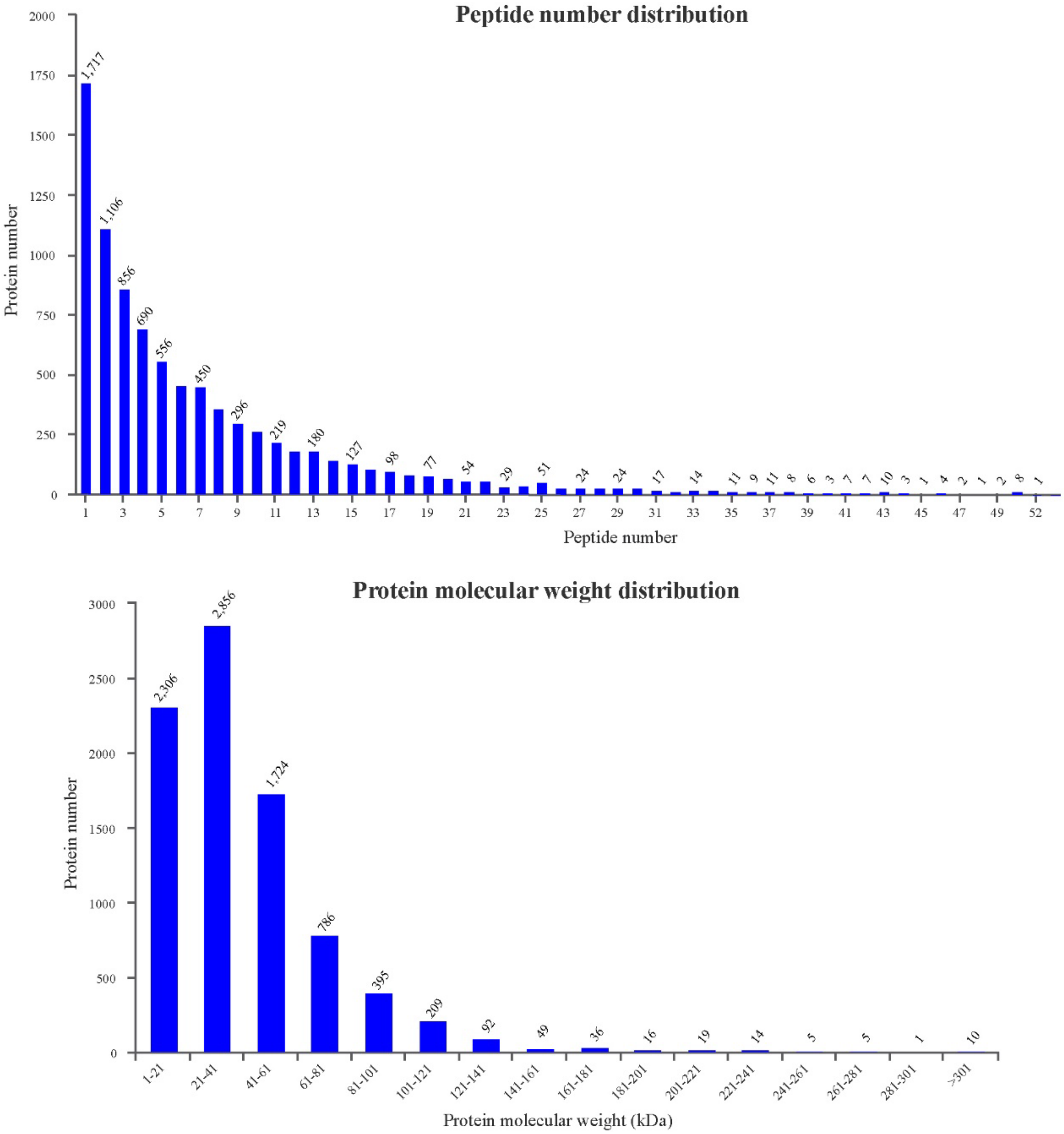

Supplementary Figure S4. Protein peptide length and size distribution.

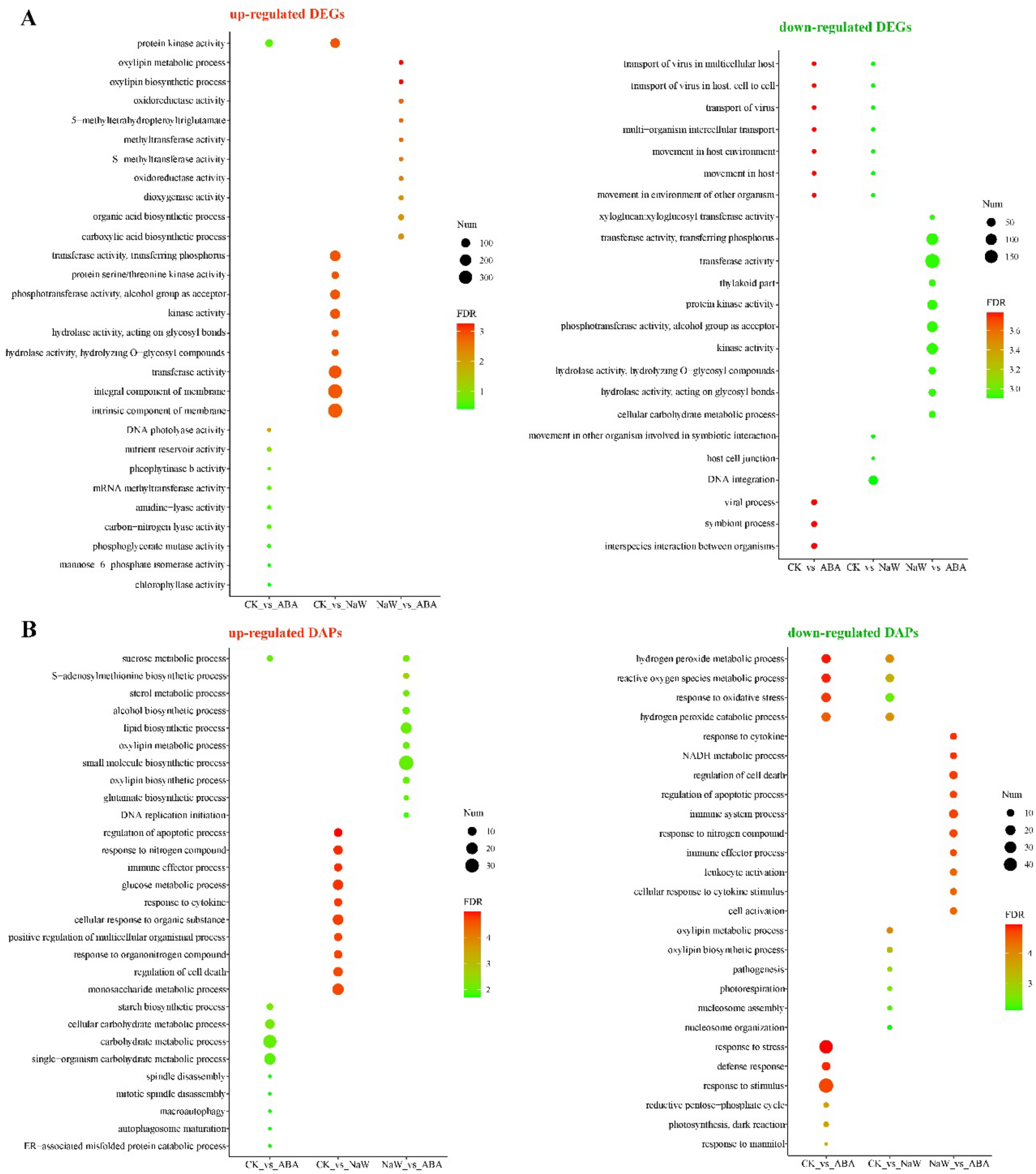

Supplementary Figure S5. GO enrichment analysis of up-and down-regulated DEGs (A) and DAPs (B) between three groups.

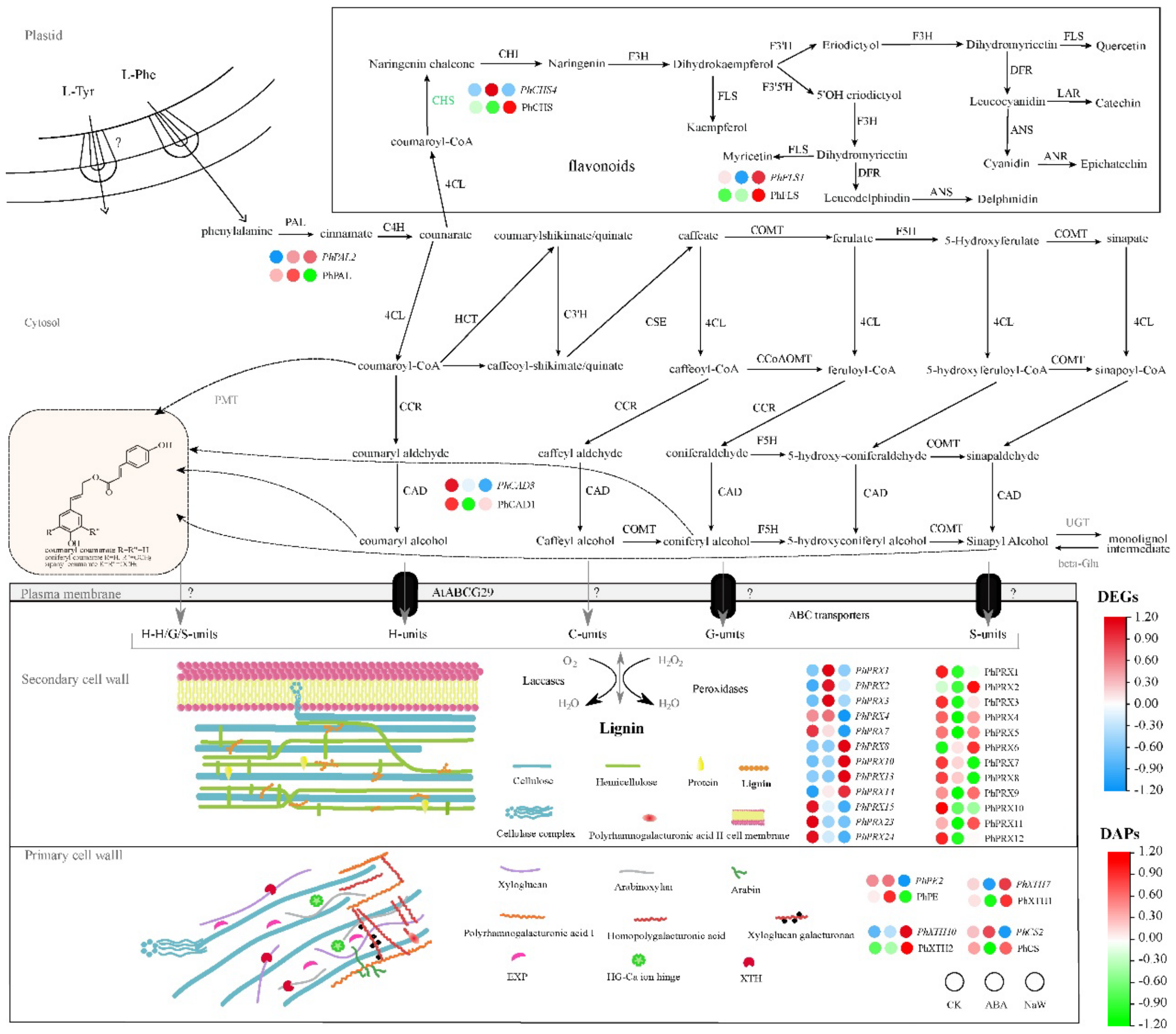

Supplementary Figure S6. Expression of DEG-DAPs in cell wall biosynthesis pathway.

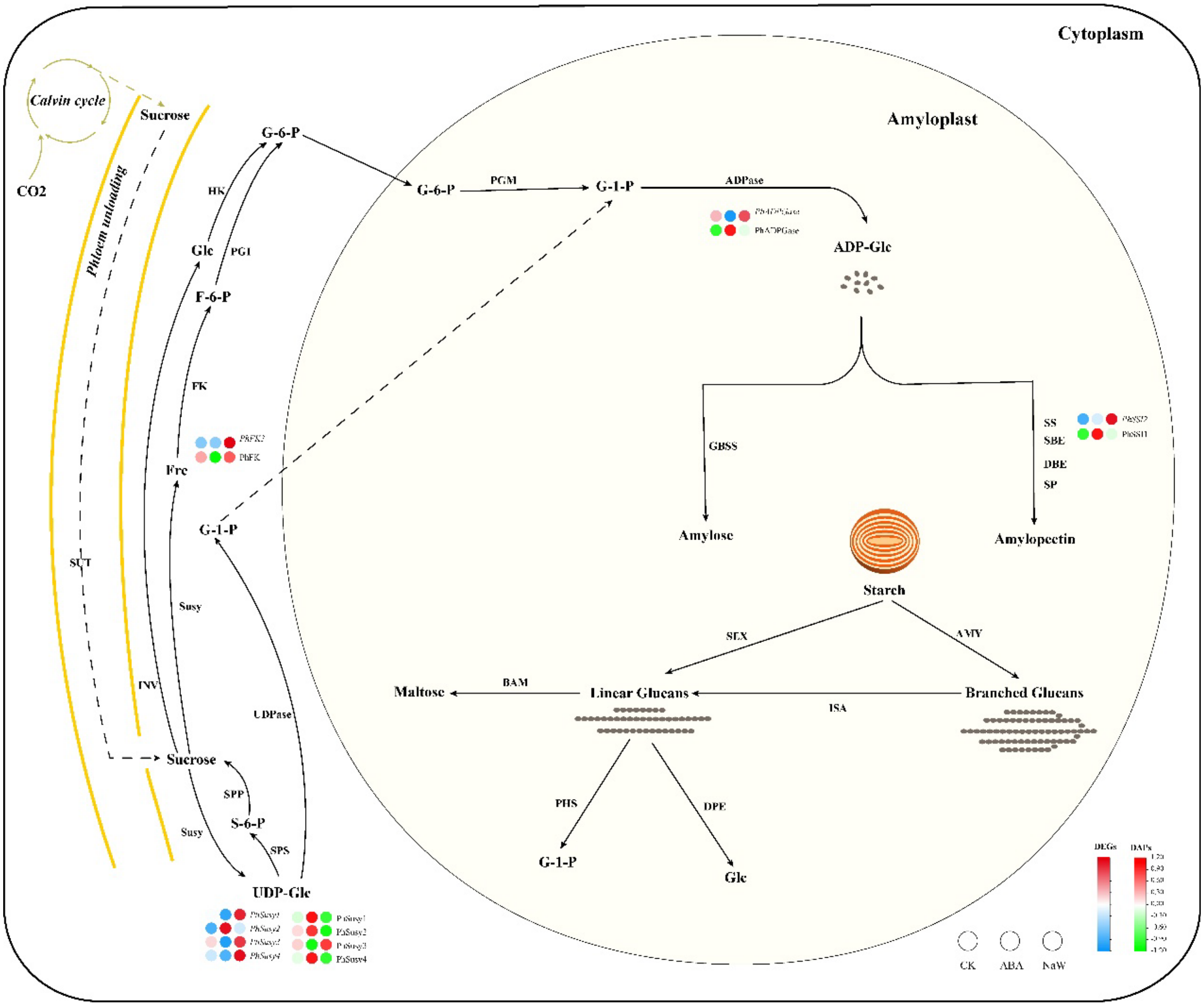

Supplementary Figure S7. Expression of DEG-DAPs in starch metabolism pathway.

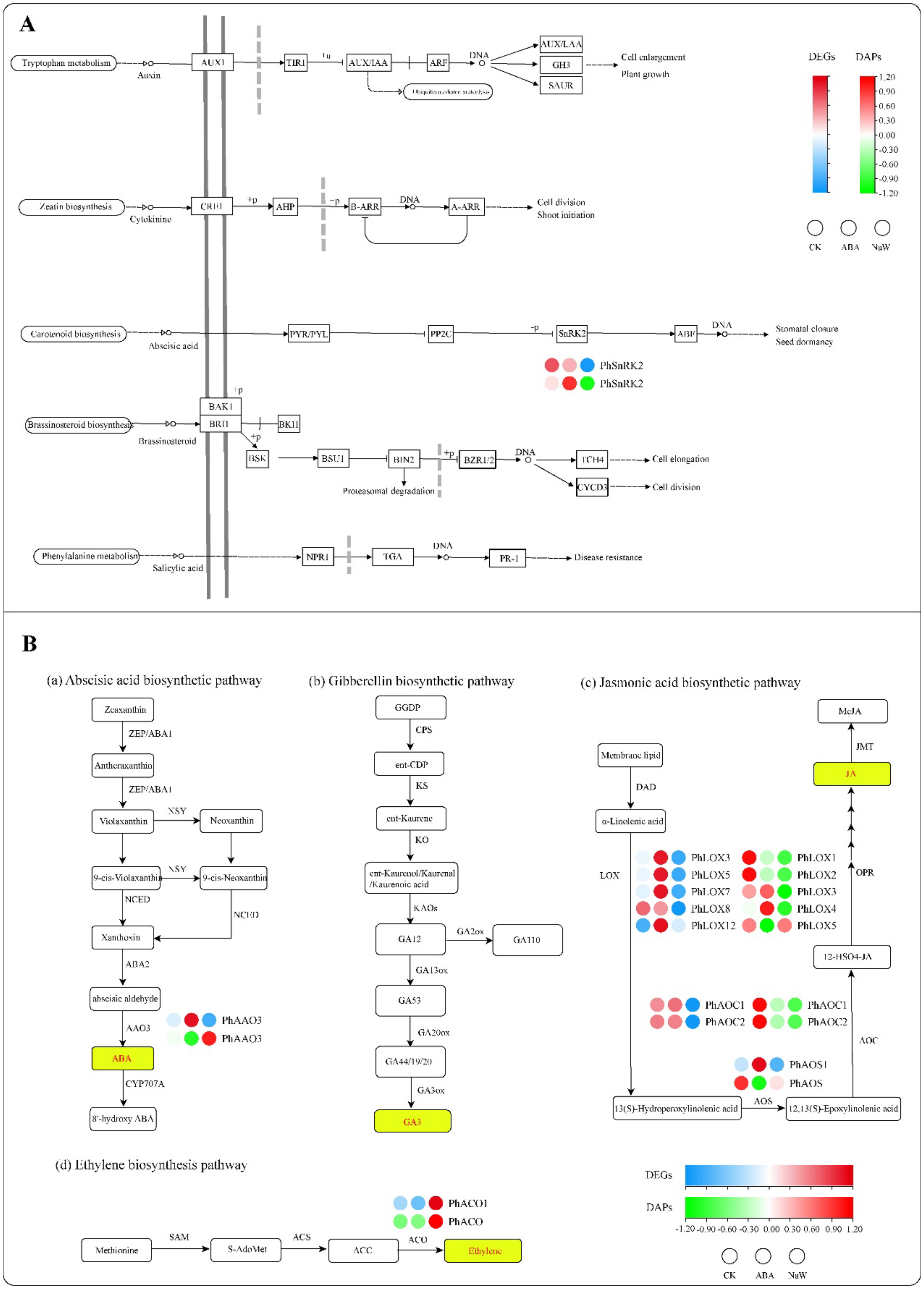

Supplementary Figure S8. Expression of DEG-DAPs in plant hormone signal transduction (A) and biosynthesis (B) pathway.
